# Supplementary material for: Are carbon emissions trading and green financial instruments synergistic? -Comprehensive quantitative research based on content analysis
Source: PLoS One. 2024 Mar 7;19(3):e0298601. doi: 10.1371/journal.pone.0298601 (PMC10919601; doi:10.1371/journal.pone.0298601)
Supplement: S1 Data — (ZIP) [file pone.0298601.s001.zip › Data/地级市Z_F工作报告.zip!/╦─┤¿/π≥╓▌╩╨/└┤╫╘╣1⁄2╓┌║┼í╛┬φ┐╦╩2╛▌═°í┐_2014.docx]

各位代表：

现在，我代表市人民政府，向大会报告工作，请予审查，并请市政协委员提出意见。

2013年工作回顾

过去一年，是泸州发展历程中不平凡的一年。在省委、省政府和市委的坚强领导下，在市人大、市政协的监督支持下，我们紧紧围绕“建设川滇黔渝结合部区域中心城市”的总体定位，牢牢把握“奋力走在全省次级突破前列”的总体要求，统筹稳增长、调结构、促改革、惠民生，实现了“着力六个突破、力争四年翻番”的良好开局。全市完成生产总值1140.5亿元，比2012年（下同）增长11.2%；规模以上工业增加值516.3亿元，增长11.3%；全社会固定资产投资866.4亿元，增长29.1%；社会消费品零售总额411.9亿元，增长14.9%；地方公共财政收入109.6亿元，增长32.4%；城镇居民人均可支配收入22821元，增长10%；农民人均纯收入8455元，增长13.3%。在此，我向大会报告，全市经济总量实现跨越升位，“九大民生工程”全面完成！

一、投资拉动，在项目建设中夯实发展后劲

一年来，我们以“项目落地见效年”为工作主题，打基础、利长远，强力实施项目攻坚，全社会固定资产投资总量跃居全省第7位，增速位居全省第2位。

项目落地见效。实施184个重点项目，完成投资617.6亿元。新开工10亿元以上的项目24个、100亿元以上的项目3个，竣工10亿元以上的项目3个。叙宜高速公路泸州段、泸州机场迁建工程开工建设，泸渝高速公路、成自泸赤高速公路泸州至自贡段建成通车。全市新增高速公路通车里程90公里，总里程达316公里。古蔺观文水库启动建设，纳溪黄桷坝水库主体工程全面完工。

要素保障有力。到位项目资金904.1亿元，增长23.1%。民间投资652.7亿元，增长37%。获批工矿废弃地复垦项目13个，实施增减挂钩项目15个，落实项目用地3.1万亩。林庄220千伏主变增容扩建工程、叙永新区110千伏输变电工程竣工投运。工业用电量增长19.5%，天然气供应量增长11.8%，成品油销售量增长4.6%。

发展后劲增强。我市列入《全国老工业基地调整改造规划（2013—2022年）》、《全国资源型城市可持续发展规划（2013—2020年）》，纳入全国二级物流园区布局城市。隆黄铁路叙永至毕节段、内泸城际铁路获得省政府支持组建项目公司，古叙煤矸石电厂、渝昆铁路、乐自泸铁路泸州段等项目前期工作加快推进。储备项目1105个，总投资达6806亿元。

二、多措并举，在市场调整中巩固泸酒优势

一年来，我们面对白酒产业的深度调整，主动作为、积极应对，千方百计稳定质量、稳定市场、稳定队伍，白酒产业在调整中发展、在发展中优化。

酒业基础进一步夯实。编制完成《泸州建设“中国白酒金三角”核心腹地总体规划》。泸州老窖“一园三基地”、二郎·名酒名镇、中国酒镇·酒庄、郎酒浓香型白酒生产基地、华夏龙窖白酒产业园、永乐酱酒园区等项目建设进展顺利。发展酿酒专用高粱61.2万亩，新建窖池5036口。酒类产品新创建中国驰名商标5件、省著名商标4件，新培育四川名牌10个。

功能平台进一步完善。国家固态酿造工程技术研究中心建设通过验收。国家酒检中心检验检测能力不断提升。国家酒类包装产品质量监督检验中心筹建顺利。四川中国白酒产品交易中心完成交易额64亿元。“泸州·中国白酒商品批发价格指数”平台发布全国白酒价格总指数36期。白酒酒庄文化服务综合标准化试点工作加快推进。

泸酒市场进一步拓展。成功举办“第十四届中国西部国际博览会白酒专业博览会”，实现交易额116.3亿元。引导酒类企业创新营销模式、拓展销售市场，泸酒中低端市场份额有效扩大。全市白酒产业实现规模以上增加值356.6亿元、增长13%，销售收入723.7亿元、增长16.8%。

三、优化升级，在结构调整中做强产业支撑

一年来，我们围绕“一产抓特色、二产抓提升、三产抓拓展”的思路，以工业为重、工业为大、工业为先，坚持调存量与扩增量并举，三次产业结构调整为13.6：60：26.4。

传统工业加速转型。化工产业加快调整步伐，空间布局进一步优化，产业链进一步延伸，天华公司1，4—丁二醇项目投入试生产。能源产业加快整合步伐，实施煤矿清理整顿和兼并重组，一次性关闭民营煤矿52处。机械产业加快技改步伐，厦门海翼·长江液压西部基础件基地一期工程完工，邦立重机异地技改项目加快建设，国机重工西南产业园、长江机械厂异地技改项目实现开工。

现代服务业快速发展。服务业增加值增长12%，增速居全省第1位。成功举办首届中国（泸州）西南商品博览会。佳乐世纪城核心功能区建设进展顺利，西南商贸城二期工程启动建设。泸州港完成货物吞吐量2707万吨、增长15.3%，其中：集装箱吞吐量20.1万标箱、增长48.9%，总量占全省的77%，“四川航运首港”地位凸显。德阳银行在我市设立分行。新增小额贷款公司13家、融资性担保公司2家。2013年末，银行业金融机构各项存款余额1410.8亿元，比年初增长16.2%；贷款余额766.8亿元，比年初增长21.8%。实现旅游总收入143亿元，增长34.9%。

现代农业增产增效。建成高标准基本农田44万亩。粮食总产量达198万吨，增长3.3%。连片发展畜牧、林竹、果蔬茶、高粱、优质稻、烤烟六大特色效益农业。新建万亩现代农业示范区17个。新培育省级农业产业化龙头企业5家，新发展农民专业合作社382家。新增国家级农业标准化示范区2个，“三品一标”产品认证达168个。四川银鸽5万吨竹纤维项目建成投产。建设“菜篮子”便民店20个。启动了第二批12个省市新农村示范片和110个新村基础设施建设，新建新农村综合体11个。

四、整体推进，在加快建设中提升城市品位

一年来，我们坚持高起点规划、高质量建设、高水平管理，推进产城互动发展，加快构建“一主四副多点”新型城镇体系，“江、山、酒、港”城市特色更加鲜明。

中心城区扩容提质。建成区面积达109.4平方公里、人口达108万人。城北绕城环线一期、龙马大道北延线道路竣工通车，五条高速公路城市进出口通道、忠山隧道、沱江六桥开工建设。城市公交专用道投入使用，建成人行天桥7座。启动实施旧城危旧房和棚户区改造项目16个。全面推进主城区“两江四岸”建设，改造长、沱两江滨江路8公里，东门口广场、单碗广场、馆驿嘴广场、龙马广场一期工程建成开放。改造提升了忠山公园、张坝桂圆林公园、护国公园等7个公园，龙透关公园一期、植物园一期、江南生态公园等9个公园和酒城乐园建成开放。城市绿地覆盖率达35.1%。创建“国家园林城市”通过验收。

县城和小城镇提档升级。泸县完成县城道路全域“白+黑”改造。合江撤县设市获得省政府同意并上报国务院审批。合江、泸县成功创建国家卫生县城。叙永县完成城市总体规划修编，县城商业综合体启动建设。古蔺县城西区建设初具规模，天立酒街二期主体工程完工。全市33个镇乡完成规划修编，5个镇列入全省第一批“百镇建设试点行动”试点镇。全市城镇化率达43.3%。

产业园区升位发展。泸州经济开发区列入“全省新型工业化产业示范重点培育基地”，成功创建“省级高新技术产业园区”。泸州酒业集中发展区、泸州经济开发区纳入全省“51025”重点产业园区发展计划，古蔺经济开发区获批筹建“四川省知名品牌创建示范区”，川渝合作园区申报为“省级小企业创业示范基地”。全市产业园区建成区面积达53.1平方公里、入驻企业1337家，完成主营业务收入1262.8亿元、增长16%。

五、统筹发展，在加压奋进中激发区县活力

一年来，我们坚持统筹规划、分类指导、突出重点，各区县自加压力、跳起摸高、竞相发展，次级突破的底部基础不断夯实。区县公共财政收入达63.6亿元，增长21%。

“三个新区”启动建设。组建了“江南新区”、“泸州临港产业物流园区”、“泸州高新技术产业园区”管委会和投融资公司。“江南新区”启动了茜草新城建设，加快了“三长”厂搬迁进度，泸合大道控制性工程开工建设，合江食品工业园、纳溪麒麟新城、江阳董允坝万亩现代农业示范园等项目建设进展顺利。“泸州临港产业物流园区”启动了核心区建设，纳福大道控制性工程、龙港路、滚装码头续建等项目开工建设，保税物流中心（B型）、进口粮食指定口岸建设和申报工作加快推进。“泸州高新技术产业园区”构建了“一区三园”模式，完成创建国家高新技术产业开发区申报工作，机械装备产业园入驻企业达70家，新能源新材料产业园成功引进北辰电力产业园等项目，泸州医药产业园入驻企业14家。

乌蒙山片区加快发展。推进“七大扶贫攻坚行动”，实施连片扶贫开发项目3个。叙永赛德水泥项目建成投产，江门20万吨竹浆纸一体化项目加快推进。观文、岔角滩、箭竹坪、西华煤矿加快建设。全国岩溶地区石漠化综合治理工程第四次省部联席会暨现场会在我市召开，“粮经复合、综合治理”、“立体农业、产村相融”、“种养结合、生态循环”、“产村一体、文旅交融”等治理模式受到高度评价。

民营经济蓬勃发展。改革工商登记制度，推行个体小额经营社区备案管理，新增市场主体2.5万户。65户民营企业纳入重点培育上市企业名单，完成宏图物流公司上市辅导工作。全市民营经济实现增加值675.1亿元，增长14.3%。

六、改革开放，在环境治理中营造比较优势

一年来，我们坚持“三为主”、突出“三重点”、强化“三主动”，在改革创新中增强动力，在开放合作中增添活力，在优化环境中争创优势。

重点领域改革迈出新步伐。农村产权制度改革稳步推进，农村土地承包经营权确权颁证工作扎实开展，集体林权制度配套改革进展顺利。调整和完善市区财政体制，实行税收属地征管，营业税改征增值税试点工作完成。深化户籍管理制度改革，全面放开城镇落户限制。人事制度、工资收入分配制度、医药卫生体制、文化体制、事业单位分类改革加快推进。转化重大科技成果12项，新增国家高新技术企业12户，新增授权专利547项。我市被评为2013年度全国科技进步先进市。

开放合作迈上新台阶。成功举办泸州·新加坡经贸合作座谈会，促成凯发集团、吉宝讯通、裕廊国际等企业在我市投资发展。主动对接大企业、大集团，新引进荣程集团白酒生产基地、航天泸州工业园等投资5亿元以上的项目74个。到位市外国内资金682.2亿元，增长32.9%。泸州港新开通“泸—汉—台”江海直达航线、泸州至上海商品汽车滚装班轮、泸州至昆明铁水联运集装箱班列。泸州机场新开通至西安、南宁、厦门、长沙航线，通航城市达10个，旅客吞吐量达44万人次、增长41%。实现进出口总额2.3亿美元，其中外贸出口2亿美元；实际到位外资5293万美元。

环境治理取得新成效。营造公平有序的建设环境，深入开展“治堵、治违、治扰、治拖”等工作，建设市场秩序明显好转。营造安定祥和的治安环境，扎实开展“打盗抢、攻毒品、治乱点”系列打击整治行动，全市刑事案件下降16.2%，社会治安状况稳定好转。营造整洁优美的城乡环境，城市风貌塑造上档升级，夜景光亮工程建设有序推进，“数字化”城市管理信息系统投入运行。营造规范有序的信访环境，全面落实属地责任，从政策层面有效化解了一批群体性信访问题。营造齐抓共管的安全生产环境，开展安全生产“五大攻坚行动”，探索创新了“十大监管方法”。强化基层基础建设，新启动14个省级“安全社区”创建单位，新建公路防撞墙和波形护栏1142公里。推进“科技兴安”，全市3154辆客车、983辆公交车安装了GPS定位系统和3G视频监控系统，96个渡口码头、76艘客渡船安装了3G视频监控系统，安全监管水平进一步提升。

七、民生优先，在惠民利民中构建和谐社会

一年来，我们坚持以人为本，加大民生投入，注重从制度层面解决民生难题，促进民生事业持续改善。财政用于民生领域的支出达175亿元，占公共财政支出的70.2%。

民生工程扎实推进。投入资金64亿元，完成119个民生项目。城镇新增就业41600人，城镇登记失业率3.1%。“五项社会保险”覆盖360万人次。新型农村合作医疗参合率达99.69%。城市低保、农村低保月人均保障标准分别提高40元和15元。开工保障性住房7198套，改造棚户区住房3056套、农村D级危房11569户。新建农村公路1381.2公里，新增农村客运班线46条。改造升级农村电网1120千米。解决农村33.4万人饮水不安全问题。完成重大地质环境治理项目9个。减少农村贫困人口73600人。

社会事业协调发展。创建“省级文明城市”通过验收。泸州大剧院建成投用。“三馆一站一书屋”全部免费开放。泸县农民演艺网创建为首批国家公共文化服务体系示范项目。新增全国文物保护单位12处。稳步推进教育均衡发展，以“三开减负三开放”为主要内容的素质教育深入实施。城区（县城）“大班额”难题逐步化解。应届高考重点本科上线人数增长43.4%，增幅居全省第1位。“川滇黔渝结合部教科城”开工建设。公共卫生保障能力不断增强。“西南医疗康健城”一期工程启动建设，市人民医院新院建设主体工程正式动工。全民健身活动蓬勃发展，竞技体育水平不断提高。奥林匹克体育公园主体育场竣工投用，张坝体育中心完成运动场地建设。人口计生“十大重点工作”全面完成，人口自然增长率4.03‰。食品药品安全专项整治工作扎实开展，餐饮服务食品安全示范工程深入推进。发展地面数字电视用户2.3万户。第三次全国经济普查顺利推进，完成了省级综合试点工作。新建新型社区服务综合体3个。完成了第九届村（居）民委员会换届选举工作。

生态环境不断优化。我市列入全国第一批水生态文明建设试点市。全面完成省上下达的节能减排指标，削减化学需氧量2.2%、氨氮3.3%、二氧化硫3%、氮氧化物4.1%。二道溪污水处理厂二期工程、垃圾焚烧发电厂开工建设。濑溪河、龙溪河、永宁河等小流域污染治理扎实推进。完成营造林46.6万亩，森林覆盖率达49.5%。创建“省级环保模范城市”通过验收。

公安“三项建设”扎实推进，维稳保安能力明显提升。“六五”普法深入开展，提供法律援助2346件。加强防灾减灾能力建设，应急避难场所全部投入使用。继续深化对口援助甘孜州稻城县和乡城县工作。对口援建宝兴县“4·20”地震灾后恢复重建取得阶段性成效。国防动员和民兵预备役建设进一步加强。民族宗教、老龄、妇女儿童、残疾人、对台、外事、侨务、消防、保密、档案、统计、气象、双拥、人防、地方志等工作取得了新成绩。

各位代表：过去一年，我们围绕提高公信力、执行力和免疫力，加快建设人民满意的服务型政府。办理人大代表建议和政协委员提案443件，办结率100%。落实政府常务会学法制度。在全省率先启动了“依法行政第三方评估”。清理规范行政权力事项6663项，调整和取消行政审批事项96项。开展城市管理相对集中行政处罚权工作，深化行政复议规范化建设。重点领域信息公开有序推进。新闻发言人制度进一步完善。政务服务体系实现四级联动，市、县公共资源交易平台一体化运行，政务服务工作连续5年获得省政府表彰。市政府门户网站改版升级，“12345”市长热线受理群众有效来电3.2万件。深入推进惩防体系建设，廉政风险防控“三早三卡”机制应用领域进一步拓展。严格执行改进工作作风、密切联系群众各项规定，扎实开展干部作风整顿，深入整治工作不具体、不落实、不负责等问题。拓展政风行风热线，加大专项治理力度。充分发挥监察、审计职能，确保了权力在有效制约和监督下规范运行、廉洁运行。

各位代表：过去一年，面对极为复杂的宏观经济形势、极为严峻的困难和挑战，在市委的科学决策、正确领导下，在市人大、市政协的有力监督、大力支持下，全市广大干部群众凝心聚力、真抓实干、攻坚克难，取得了来之不易的成绩。在此，我代表市人民政府，向关心、支持、推动泸州发展的全市人民、驻泸部队官兵和各界人士，表示衷心感谢并致以崇高的敬意！

各位代表：过去一年，受国际国内市场深度调整、经济下行压力加大、经济结构性矛盾凸显等因素影响，我市部分经济指标增速未能实现市七届人大三次会议确定的预期目标。同时，全市经济社会发展还面临不少困难和问题：产业结构不尽合理，产业支撑不够有力；改善民生任务繁重，全市还有41万贫困人口，就业、教育、医疗、住房等领域关系群众切身利益的问题亟待解决，促进城乡居民持续增收的难度加大；社会治理面临不少新的情况，安全生产压力较大，社会治安形势依然复杂；政府自身建设与人民群众的期望还有一定差距，服务质量、服务效率还需进一步提高，廉政建设还需进一步加强。对这些困难和问题，我们将采取有力措施加快解决。

2014年工作安排

今年是在新的起点上全面深化改革的开局之年，是“着力六个突破、力争四年翻番”的关键一年。做好今年政府工作，要用改革统领经济社会发展全局，全面落实中央、省委、市委经济工作和城镇化工作会议部署，坚持“稳中求进、进中求好、好中求快”的工作基调，突出“产业发展突破年”的工作主题，坚定“凝心聚力、加快发展”不动摇，坚定“着力六个突破、力争四年翻番”不动摇，加快转方式调结构，加快建设区域中心城市，加快提升开放合作水平，着力保障和改善民生，促进经济持续健康发展、社会和谐安全稳定。

全市经济社会发展的主要预期目标是：生产总值比2013年（下同）增长10%，规模以上工业增加值增长10%，全社会固定资产投资增长19%，社会消费品零售总额增长12%，地方公共财政收入增长12%，城镇居民人均可支配收入增长11%，农民人均纯收入增长12%。

围绕上述目标，重点做好以下工作：

一、紧扣突破主题，推动产业结构优化升级

大力调整工业结构。推动白酒产业领先发展、率先发展。加快泸州老窖“一园三基地”、二郎·名酒名镇、中国酒镇·酒庄等项目建设。力争国家酒类包装产品质量监督检验中心通过验收，积极争取全国白酒酒庄文化标准化技术委员会落户泸州。加强酒类企业梯度培育，加大泸酒品牌保护力度。引导企业调整产品结构，扩大泸酒中低端市场份额。办好“中国国际酒业博览会”，不断提升泸酒影响力和知名度。加快推动化工、能源、机械产业转型发展。推进北方公司有机硅产品下游生产线项目建设，做好中海沥青投资新建项目前期工作。确保观文煤矿投产运营，加快岔角滩、箭竹坪、西华煤矿建设，推进古叙煤矸石电厂、石屏二矿前期工作。大力发展新能源汽车产业。推动机械产业向高端装备制造产业升级，重点发展大型工程机械、石油钻采装备、航空航天装备、节能环保装备。

加快推进农业现代化。稳定粮食播种面积，确保粮食总产量达195万吨。提高农业产业化水平，加强农业产业示范基地建设，推动特色农业规模化发展。新建现代林业基地20万亩。大力培育新型农业经营主体，发展市级以上农业产业化龙头企业10家，建设规范化农民专业合作社20个，发展家庭农场20个。加快发展农产品精深加工，加强农产品产销衔接。提高主要农作物耕种收农机化综合水平。推进第二批12个省市新农村示范片建设，新建新农村综合体9个，启动50个幸福美丽新村建设试点。

全面提升服务业发展水平。完成《主城区商业网点规划》修编，推进佳乐世纪城、西南商贸城二期建设。启动泸州市农产品批发市场建设，加快规划和推进公益性农贸市场建设试点。完善物流产业发展综合体系，培育和引进大型物流企业。拓展泸州港经济腹地，力争集装箱吞吐量达到25万标箱，提升“四川航运首港”地位。积极引进金融机构在我市设立分支机构和区域性总部，大力发展资本市场，推动优质企业上市融资，规范发展小额贷款公司、融资性担保公司等准金融机构。推动文化遗产保护与旅游产业融合发展。加快玉蟾山温泉度假区、凤凰湖旅游开发等项目建设，力争张坝、天仙硐创建为国家4A级旅游景区。力争创建“全省旅游标准化示范试点市”通过验收。完成旅游总收入165亿元。积极培育电子商务、会展经济、文化创意等新兴服务业。

二、注重以人为本，推动新型城镇化健康发展

建设畅通宜居城区。加快建设沱江六桥、忠山隧道工程，力争开工建设长江二桥、长江六桥；加快城市环线及连接线工程建设，完成城南、黄舣、石洞、胡市、城西、纳溪、方山、龙港路八个城市进出口通道建设，努力缓解城市交通拥堵。推进旧城危旧房和棚户区改造，新启动中心城区10个改造项目。深入推进主城区“两江四岸”建设，建成开放长江生态湿地公园一期、玉带河湿地公园二期、东岩公园等城市公园，完成杨桥湖城市公园的规划设计。实施环境质量提升、扬尘污染治理等专项行动，营造整洁优美的城乡环境。启动第六次“国家卫生城市”复审工作。

优化完善城镇体系。完成《泸州市城镇化发展规划》。优化城镇空间布局，加快构建城市发展走廊，推动各县城与中心城区同城化发展。提高城镇建设用地集约化程度，增强县城综合承载能力。优化调整行政区划，加快合江撤县设市工作，支持有条件的镇改设街道办事处。加强重点镇和中心村建设，开展“全国重点小城镇”、“中国历史文化名镇”、全省第二批“百镇建设试点行动”试点镇申报工作。确保全市城镇化率提高1.6个百分点。

推进农业转移人口市民化。深化户籍管理制度改革，加强城镇实有人口服务管理。引导和鼓励农业转移人口就近就地落户，促进转户人口向主城区、县城和中心镇有序集聚。实施“农民工住房保障行动”，建设公共租赁房4000套，定向分配农民工公共租赁房600套。新开工保障性住房2500套。稳步推进城镇常住人口基本公共服务全覆盖，完善农民工养老、失业、医疗等社会保障体系。实施农民工职业技能提升计划，保障农民工随迁子女平等接受义务教育。注重产城融合，提升城镇吸纳就业能力。

三、强化落地见效，推动项目投资快速增长

加快交通基础设施建设。确保成自泸赤高速公路泸州至赤水段建成通车。加快建设叙古高速公路、叙宜高速公路泸州段、叙大铁路、泸州机场迁建工程。力争G8515荣昌（川渝界）至泸州高速公路开工建设、叙威高速公路泸州段纳入省高速公路路网规划。推进隆黄铁路叙永至毕节段、渝昆铁路、内泸城际铁路、泸州至自贡大件公路前期工作。

加快城镇基础设施建设。加快建设二道溪污水处理厂二期工程，启动纳溪污水处理厂扩建工程，开工建设城东、泰安污水处理厂，推动截污干管工程建设。力争启动南郊、北郊、茜草水厂扩建工程和纳溪水厂新建工程。加快建设垃圾焚烧发电厂，开工建设城南垃圾压缩中转站，做好城西、城北垃圾压缩中转站前期工作。开工建设泰安220千伏、邻玉110千伏输变电工程。加大供气、通讯、光纤等基础设施建设力度。

加快农村基础设施建设。新建通乡公路300公里、通村公路210公里。实施重点骨干水源工程，加快建设合江锁口水库、叙永倒流河水库、古蔺观文水库。大力实施中低产田和坡耕地改造，加快农村电网改造、土地综合整治和高标准基本农田建设。加快农村环境基础设施建设，完善农村生活垃圾设施。

四、加强分类指导，推动县域经济发展壮大

统筹“三个新区”发展。围绕“两化”互动、“三化”联动，加快建设“江南新区”。推进南部新城起步区、茜草新城、合江新城建设，启动江南生态城核心区建设。积极打造纳溪都市农业样板区、江阳董允坝万亩现代农业示范园、合江大桥酒庄经济实验区。力争启动纳溪转型发展示范园区建设，推动泸州酒业集中发展区创建全国生态工业园区。加快合江川渝合作示范区建设，启动川黔、川滇合作示范区建设。围绕港产城一体化发展，加快建设“泸州临港产业物流园区”。推进纳福大道、龙港路、成自泸赤高速公路与泸州港互通连接线、滚装码头续建等项目建设，启动实施神仙桥码头二期工程，力争泸州港保税物流中心（B型）、进口粮食指定口岸申报成功。围绕培育壮大新兴产业，加快建设“泸州高新技术产业园区”，争创国家高新技术产业开发区。依托机械装备产业园，加快建设国机重工西南产业园、长江机械厂异地技改等项目；依托新能源新材料产业园，建成北辰电力产业园项目一期，跟踪引进电动汽车电池等项目；依托泸州医药产业园，加大招引力度，确保新入驻医药企业3家以上。

加快扶贫攻坚步伐。以乌蒙山片区为主战场，深入开展“七大扶贫攻坚行动”，提升贫困地区自我发展能力。坚持开发式扶贫，创新工作机制，实施连片扶贫开发项目3个、整村推进项目25个，完成农村扶贫解困73800人。积极帮助叙永、古蔺落实少数民族地区待遇县相关政策。

破解区县发展难题。坚持市区县一体化发展，大力培育县域经济。着力盘活存量，不断提高节约集约用地水平。积极争取用地政策，稳妥推进政策性用地指标跨区县有偿流转。搭建投融资平台，拓宽项目资金筹集渠道。严格控制新增污染，保障建设项目环境容量需求。探索多渠道融资，放宽民间资本市场准入领域和条件，扶持中小微企业，助推民营经济健康发展。

五、突出重点领域，推动改革创新取得实效

加快推进经济体制改革。完善国有资产管理体制，探索组建国有资本运营公司。推进公司注册资本登记制度改革，推广个体小额经营社区备案管理制度。深化金融改革创新，做好市商业银行增资扩股、农村信用社改制工作。深化财税体制改革，探索合理划分市与区县事权和支出责任。推进预算管理制度改革，加强政府债务管理，防范和化解财政风险。深化农村产权制度改革，稳步推进土地管理制度改革，基本完成农村“五种权属”的实测确权登记工作。开展农村土地流转收益保证贷款试点，探索发展土地股份合作社。

积极推进社会事业改革。巩固医药卫生体制改革成果，深化公立医院、基层医疗卫生机构综合改革。适时启动实施“单独两孩”政策。制定统一的城乡居民基本医疗保险办法，实施城乡居民大病保险。扩大养老保险参保覆盖面，促进城乡养老保险制度转移衔接。深化素质教育综合改革，统筹城乡义务教育资源均衡配置，破解城区（县城）“大班额”和幼儿园入学难问题。推进文化体制改革。继续完善人事制度、收入分配制度改革。

稳步推进行政体制改革。推进市县政府部门机构改革，全面实行机构编制实名制管理。深化行政审批制度改革，建立健全动态调整机制。优化建设工程项目并联审批，简化审批环节，提高审批效率。完善事业单位分类改革配套政策。启动行业协会商会与行政机关脱钩工作。推进政府采购标准化建设。

切实推进创新能力建设。提高科技创新驱动能力，积极实施“技术创新示范行动”，加快科技成果产业化、市场化。加大国家企业技术中心、高新技术企业、创新示范企业培育力度，加强知识产权保护。推动“国家商标战略实施示范城市”创建工作，力争创建“全国质量强市示范城市”通过验收。探索人才引进新机制，加大人才培养力度，关心各类人才成长。

六、坚持招大引强，推动开放合作效益提升

增强招商引资实效。整合招商资源，发挥区县、园区、企业主体作用，提高招商针对性和实效性。坚持选商引资，立足全球市场，围绕产业链、供应链、价值链招大引强，突出招引工业、服务业项目和农业产业化龙头企业。确保引进到位市外国内资金增长10%。

提升对外合作层次。深化与新加坡等国家的交流合作。抢抓国家建设长江经济带、丝绸之路经济带机遇，做好泸州水运口岸申报国家开放口岸工作，深化与长江流域港口城市、中国—东盟自由贸易区合作。巩固“泸—汉—台”江海直达航线，争取开通泸州至武汉至韩国等近洋航线。推动赤水河流域保护与开发，探索建立长江上游生态屏障和扶贫开发试验区。主动对接成渝“双核”，推动川南经济区一体化进程。

促进开放型经济发展。坚持“走出去”与“引进来”相结合，提高开放型经济质量。丰富利用外资方式，促进外资股权投资和创业投资。优化提升对外贸易，加快培育重点出口园区和企业基地。鼓励企业开展对外承包工程，建设外派劳务基地。

七、办好为民实事，推动民生事业持续改善

实施民生工程。确保公共财政预算用于民生支出的比例达70%。推进就业促进、扶贫解困、教育助学、社会保障、医疗卫生、百姓安居、基础设施、生态环境、文化体育等重点民生工程。落实各项就业促进政策，推进“省级创业型城市”创建工作。城镇新增就业3.5万人，城镇登记失业率控制在4%以内。确保“五项社会保险”总覆盖650万人次。改造棚户区住房23591户、农村危房14331户。加大地质灾害防治力度，推进地质灾害避险搬迁安置工程建设。力争解决农村25万人饮水不安全问题。加大食品药品安全专项整治力度。保持物价总水平基本稳定。

发展社会事业。提高教育教学质量，开展“教育均衡发展合格乡镇”创建，做好国家中小学素质教育综合评价改革实验区工作。加快建设川滇黔渝结合部医教园区。实施重点文化惠民工程，加快“幸福美丽乡村（社区）文化院坝”建设。加大对历史文化遗产的挖掘、保护和利用力度。启动《川南区志》编修工作。健全城乡医疗卫生服务体系，提高公共卫生服务均等化水平，完善市级妇幼保健功能。加快建设西南医疗康健中心，推动市人民医院和市中医医院新院建设。完善社会养老服务体系，提高养老服务水平。全面建成奥林匹克体育公园，力争完成江南体育生态园主体框架，启动白招牌全民健身广场建设。实施人口计生“六大行动”，促进人口长期均衡发展。完成广播电视“村村通”建设，探索建立农村应急广播体系。继续做好第三次全国经济普查工作。加强国防动员、国防后备力量和民兵预备役建设。做好双拥、民族宗教、妇女儿童、残疾人、保密、统计、档案、人防、气象等工作。

改善生态环境。加强天保工程建设，巩固和推进退耕还林工程，严格森林资源保护。加大石漠化、地质环境恢复、土壤污染修复和水土流失治理力度。推进生态县、生态乡镇、生态村、生态小区建设。控制和减少农村面源污染，实施小流域综合治理。推进乡镇集中式饮用水源保护。强化节能减排工作，加大环境空气污染治理力度。

八、加强社会治理，全力维护社会和谐稳定

强化安全生产监管。完善落实安全生产“一岗双责”、“一票否决”制度。不断创新安全生产监管方式，加强“科技兴安”，强化基层安全管理。动态排查和治理安全隐患，全面加大安全整治力度。强化安全生产“五级监管”，把各项措施落实到基层一线和操作者层面。扎实开展“道路交通安全综合整治攻坚年”行动，深入开展“打非治违”专项斗争，坚决扭转安全生产工作被动局面。

改进社会治理方式。创新群众工作方法，进一步畅通和规范群众诉求表达渠道，健全重大决策社会稳定风险评估机制，注重运用法治思维和法治方式解决信访突出问题。深入实施“六五”普法规划，健全法律援助制度。完善流动人口和特殊人群的服务管理。实施邻里中心试点项目，建设新型社区服务综合体。加强网络社会管理，推进网络依法规范有序运行。强化应急管理，提高防灾减灾能力。

巩固深化平安建设。扎实推进公安改革创新，不断提升能力水平。完善治安防控体系，构建街面巡逻防控、城乡社区村庄防控、单位内保防控、行业场所防控、视频技术防控“五张网络”。继续实施社会治安专项行动，严打多发侵财犯罪、集中整治毒品问题，深化整治治安乱点，促进社会治安更好、群众安全感更强。

各位代表：做好今年工作，对政府自身建设提出了新的更高要求，我们将更加注重依法行政、务实勤政、清廉从政、为民施政，加快建设法治政府、效能政府、廉洁政府。

推进依法行政。坚持依法治市，以法治思维和法治方式履行职能。自觉接受市人大及其常委会的法律监督、工作监督和市政协的民主监督，高度重视司法监督、舆论监督和社会公众监督。加强与民主党派和人民团体的联系。支持工会、共青团、妇联等群团组织开展工作。推动领导干部学法经常化，充分发挥政府法律顾问团作用，在政府部门普遍建立法律顾问制度。全面完成城市管理相对集中行政处罚权工作，启动相对集中行政复议权工作。实施“开放式决策”。规范行政执法自由裁量权行使。完善政府信息公开和新闻发言人制度。

提高行政效能。进一步简政放权，加快转变政府职能。强化政务服务体系建设，加快全程电子化招投标。优化公共资源交易平台，完善流程化管理机制。推动电子政务平台升级，推进行政权力依法规范公开运行。提升政府网站服务水平，办好“12345”市长热线。全面推进政府绩效管理，优化目标绩效考核。扎实开展党的群众路线教育实践活动，坚决纠正“四风”，深入整治工作不具体、不落实、不负责等问题，推进作风建设制度化、常态化。

强化廉政建设。深化惩防体系建设，注重源头预防腐败。开展专项治理，及时有效解决群众反映强烈的热点难点问题。拓展延伸廉政风险防控“三早三卡”机制，加强对权力运行的监督制约。厉行勤俭节约，严格控制“三公”经费支出，坚决反对铺张浪费。严格执行部门预算、政府采购、公开招投标制度，加强工程建设、公共资源交易等重点领域监督，强化行政监察、财政监督和审计监督，把权力关进制度的笼子里，让权力在阳光下运行。

各位代表：时代赋予重任，奋斗铸就辉煌。我们坚信，任何困难都改变不了泸州人民追求美好生活的强烈愿望，任何挑战都阻挡不了我们凝心聚力、加快发展的坚定步伐。让我们在市委的坚强领导下，在市人大、市政协的监督支持下，紧紧依靠全市人民，同心同德、和衷共济、奋勇争先，为“着力六个突破、力争四年翻番”，为在全省率先实现次级突破、率先实现全面小康而努力奋斗！
